# Fermented Soybean Meal and Its Application in Animal Husbandry: A Review

**DOI:** 10.3390/microorganisms14030691

**Published:** 2026-03-19

**Authors:** Lina Tokuna Mulalapele, Lei Xu, Dongxu Ming, Yanpin Li, Wenjuan Sun, Xilong Li, Yu Pi

**Affiliations:** Key Laboratory of Feed Biotechnology of Ministry of Agriculture and Rural Affairs, Institute of Feed Research, Chinese Academy of Agricultural Sciences, Beijing 100081, China; mulalatokuna@gmail.com (L.T.M.); xlei0611@163.com (L.X.); mingdongxu@caas.cn (D.M.); liyanpin@caas.cn (Y.L.); sunwenjuan@caas.cn (W.S.)

**Keywords:** protein diet, soybean meal, microbial fermentation, poultry, livestock

## Abstract

Soybean meal (SBM) is a foundational protein source, but its industrial application is constrained by a complex matrix of anti-nutritional factors (ANFs). This review provides a critical synthesis of the biochemical transition from raw SBM to fermented SBM (FSBM), focusing on the synergistic mechanisms of fungal and bacterial co-fermentation. We identify that the efficacy of FSBM is primarily driven by the microbial proteolysis of glycinin into low-molecular-weight bioactive peptides (<1000 Da). These peptides serve as the primary drivers for improved intestinal morphology (increased villus height) and the modulation of the gut microbiota, providing a mechanistic basis for reported probiotic effects. Furthermore, we establish that the 5–10% improvement in the feed conversion ratio (FCR) documented for swines mathematically offsets the processing premium of fermentation. However, critical gaps remain in the standardization of solid-state fermentation (SSF) protocols, specifically regarding the selection of fungal (*Aspergillus*) and bacterial (*Bacillus* or *Lactobacillus*) strains, whose distinct metabolic pathways significantly diversify the functional profile of the resulting FSBM.

## 1. Introduction

Soybean (SB) is a common legume, among the most extensively cultivated oilseed crops globally, and serves as one of the most economical and plentiful sources of vegetable protein utilized in meals and dietary supplements [1]. Proximate analysis reveals a composition of approximately 40% protein, 35% carbohydrates, 20% lipids, 5% minerals, and 10% moisture; additionally, it contains diverse bioactive constituents, including saponins, flavonoids, isoflavones, vitamins, and phenolic compounds [2,3]. While industrial processing primarily targets oil extraction (18–20% of the bean), the residual crushed soybean meal (SBM) is the foundational protein staple for global livestock production [4,5,6]. Despite its balanced amino acid profile, rich in lysine, threonine, and tryptophan, the utility of SBM in animal husbandry is significantly constrained by a complex matrix of anti-nutritional factors (ANFs), including trypsin inhibitors (TIs), lectins, and allergenic proteins that impair nutrient bioavailability and digestive efficiency [7,8].

Microbial fermentation represents a robust bioprocessing strategy to mitigate these ANFs and enhance the suitability of SBM for sensitive livestock species [9]. This process leverages the metabolic synergy between microorganisms; while fungal species (*Aspergillus*) catalyze the degradation of structural fibers and recalcitrant carbohydrates, bacterial species (*Lactobacillus* and *Bacillus*) drive rapid acidification and the proteolysis of globulins into bioactive peptides [10,11,12,13]. Collectively, these transformations optimize intestinal homeostasis, stimulate feed intake, and support systemic immune modulation [14,15,16].

Despite these documented benefits, significant knowledge gaps remain regarding the industrial scaling of fermented SBM (FSBM), specifically concerning the synergy of multi-strain inoculants and the bio-economic feasibility of replacing conventional SBM in large-scale operations [17,18,19,20,21]. Therefore, this review provides a critical synthesis of the biochemical transitions during fermentation, elucidating how specific microbial interactions optimize the nutritional and functional quality of SBM. Furthermore, we evaluate the metabolic breakthroughs and industrial challenges of utilizing FSBM in poultry and livestock to provide a framework for future research and sustainable scaling.

### Methodology

To ensure a comprehensive and high-quality synthesis of FSBM research, a literature search was conducted across the Web of Science, Scopus, Google Scholar, and PubMed databases, covering publications from 2015 to 2026. The search utilized primary keywords, including “Solid-State Fermentation”, “Soybean Meal”, “Fermented Soybean Meal”, “Anti-nutritional Factors”, “Bioactive Peptides”, and “Livestock Growth Performance”.

Articles were screened based on the following inclusion criteria: (1) publication in peer-reviewed journals indexed in Q1 or Q2 quartiles; (2) focus on microbial-derived (fungal or bacterial) fermentation rather than chemical hydrolysis; and (3) provision of quantitative performance metrics (e.g., FCR, ADG, or histomorphological data). Approximately 190 documents were critically examined, with preference given to meta-analyses and recent clinical trials that established mechanistic links between fermentation kinetics and animal physiology. This rigorous selection process ensures that the findings discussed herein represent the current state of high-impact research in the field.

## 2. Nutritional and Anti-Nutritional Profile of Soybean Meal (SBM)

**Figure 1:**
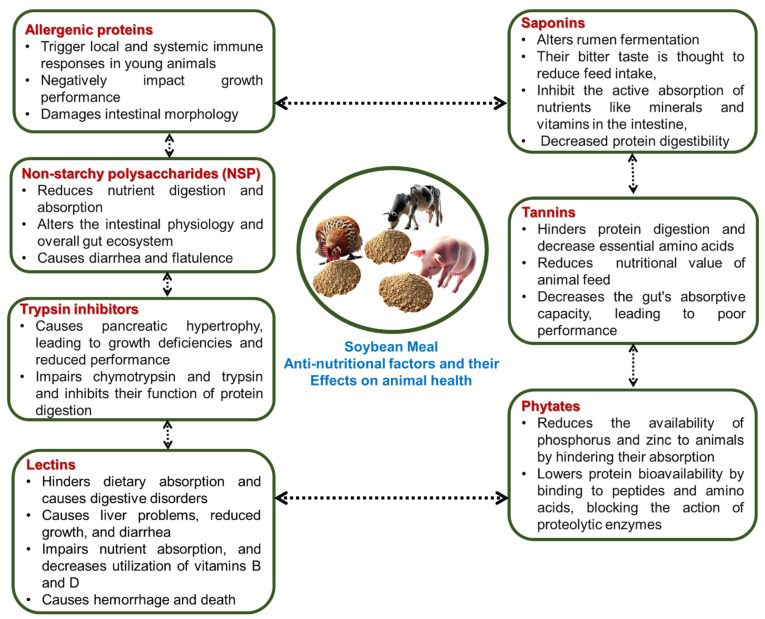
Anti-nutritional factors in soybean meal and their health effects on animals.

Soybean meal is globally recognized as the benchmark protein source for animal nutrition, primarily due to its high crude protein content (44%) [22] and amino acid profile characterized by a high lysine digestibility [23,24,25]. Despite these benefits, SBM contains a complex matrix of ANFs that significantly limit its inclusion in diets for young animals or sensitive species [26]. The nutritional density of SBM is characterized by a robust protein and lipid profile (Table 1); however, its usefulness is constrained by a diverse array of ANFs (Figure 1) that impair digestion and trigger immune responses in monogastrics.

It is rich in non-starchy polysaccharides (NSP) and oligosaccharides (raffinose and stachyose) that increase digesta viscosity and cause osmotic distress in monogastric animals [27]. Furthermore, bioactive components like saponins, tannins, and phytates interfere with mineral bioavailability and enzyme activity [28], while TIs (Kunitz and Bowman-Birk) and lectins directly impair protein hydrolysis and intestinal absorption [28,29].

The most critical limitations, however, are the allergenic proteins (glycinin and β-conglycinin) [30]. These antigens trigger systemic immune responses, upregulating pro-inflammatory pathways, nuclear factor-kappa B (NF-κB) and mitogen-activated protein kinases (MAPK), which enhances the phosphorylation of these proteins [31]. While conventional thermal processing can reduce heat-labile factors like TIs, it may inadvertently trigger Maillard reactions, which are non-enzymatic browning reactions that occur during extreme heating, where amine groups bind to the carbonyl groups of reducing sugars, ultimately decreasing the nutritional value of soybeans [32]. Consequently, microbial fermentation has emerged as the premier bioprocessing strategy to neutralize this diverse array of ANFs while simultaneously improving nutrient density.

## 3. Fermented Soybean Meal

Fermentation is one of the ancient methods of food preservation and processing [33] and can enhance the functional and nutritional qualities of the raw product [34]. Researchers have extensively utilized it to boost nutrient bioavailability [35] and decrease the presence of ANFs [36] in soybeans. Several studies [36,37,38] have established that the fermentation process is effective in breaking down anti-nutritional properties in SBM, thus increasing its potential use in various processed soybean products. Multiple microorganisms are employed to ferment SBM for nutritional improvement. The utilization of bacteria and molds facilitates the process of fermentation. However, the nutritional quality and fermentation conditions of the resulting FSBM can differ based on the type of microorganism employed. *Aspergillus* is the most prevalent species due to its enzyme-producing ability. It is capable of producing enzymes such as amylase, hemicellulase, tannase, lipase, hydrolases, proteases, and pectinases. Moreover, utilizing *Aspergillus oryzae* for fermentation can improve the nutritive quality of SBM. This highlights the possibility of using FSBM with reduced levels of TIs and higher concentrations of small-sized peptides as a viable replacement for proteins in meals of young animals [39]. *Aspergillus niger* was used in another study to examine the nutritional content and phytase activity of FSBM [40]. However, *Bacillus* and *Lactobacillus* spp. have been utilized in most studies. For instance, *Lactobacillus plantarum* FJAT-13737 and *Bacillus subtilis* FJAT-4842 were used to enhance the nutritional quality and minimize the likelihood of contamination in solid-state fermentation (SSF) of SBM [41]. Isolated *Bacillus* strains were utilized for SSF, which improved the functional properties of SBM [42].

### 3.1. Fungal Fermentation of Soybean Meal (FSBM)

Different species of the *Aspergillus* genus have been utilized to ferment SBM, such as *Aspergillus ficuum*, a mixture of *Aspergillus ficuum* and *Aspergillus niger* [43], *Aspergillus niger* [13], and *Aspergillus oryzae* [44]. The study by Jazi et al. and Yasar et al. highlighted the significance of fermentation in enhancing dietary quality, decreasing anti-nutritional factors, paving the way for future research on FSBM, and improving animal husbandry [43,45]. Notably, the positive impacts of fungi-based fermentation are well-established in the literature [8,46].

A combination of *Aspergillus oryzae* and *B. subtilis* decreased the saponin, tannin, and phytic acid of SBM drastically and increased the 2,2-Diphenyl-1-picrylhydrazyl (DPPH) antioxidant activity and total phenolic compound concentration [47]. Fermentation using *Aspergillus oryzae* resulted in the breakdown of phytate; oligosaccharides were eliminated, and the non-reducing polysaccharide content was reduced by 67%. After 72 h, fungal fermentation successfully reduced the amount of TI and phytate in soybean hulls, while significantly increasing moisture (7.07–8.23%), lipid (3.58–21.04%), and protein content (4.98–22.42%) [48]. In addition to degrading anti-nutritional factors, fungal fermentation enhances the nutritive quality of feed by boosting crude protein (CP) and crude fat levels [40]. The reduction in carbohydrate content partly contributes to the surge in fat and protein content in the fermentation process. Fermentation in SBM results in a significant increase in small-sized peptides due to the breakdown of long-chained proteins [49]. According to a previous study using edible mushrooms that compared unfermented SBM to FSBM, the results showed an improved protein profile, enhanced antioxidant capacity (as measured by DPPH and ABTS^+^ assays), and better processing potential of the FSBM [46]. Additionally, fungal solid-state fermentation can convert glycosides into aglycone isoflavones, which are more readily absorbed.

### 3.2. Bacterial Fermentation

*Bacillus* species have traditionally been utilized in producing fermented soy-based foods [8]. Like fungal fermentation, bacterial strains are also proficient in breaking down various ANFs in SBM, such as TIs, tannins, raffinose, etc. Additionally, *Lactobacillus* spp. are commonly employed bacterial strains for fermenting SBM [50,51]. *Lactobacillus plantarum* is a kind of lactic acid bacterium that, when used in fermentation, leads to protein hydrolysis and a greater release of free amino acids. This results in FSBM having significantly higher total free amino acid content than SBM [52]. These results align with the findings of Yan’s group [7]. When *B. subtilis* ferments SBM, it increases lysine and threonine levels, essential amino acids limited in swine diets. This reduces the need for synthetic amino acid supplementation. Fermentation also improves protein concentration, profile, and amino acid content, making it beneficial for weaned piglets [53].

Bacterial fermentation reduces protein size, similar to fungal fermentation, which enhances nutritional quality by removing ANFs in SBM [37]. Post-fermentation, in vitro trypsin digestibility improves, enhancing the nutritional and functional qualities compared to unfermented SBM [54,55]. Bacterial strains can boost antioxidant activity during fermentation. The high levels of amino acids such as serine, histidine, lysine, and valine post-fermentation are believed to contribute to this enhanced antioxidant property [56]. Additionally, following fermentation, the concentration of phenolic compounds rises, leading to greater antioxidant activities and metal-chelating functions [57,58]. Fermenting SBM with *B. subtilis* produces FSBM with higher CP content and lower TI levels compared to the unfermented SBM [58]. These results demonstrate that the enzymes involved in SBM fermentation heavily influence the complex process, and the choice of organism for fermentation plays a critical role in determining the nutritional composition of the resulting FSBM.

## 4. Industrial Considerations: Strain Selection, Limitations, and Economic Viability

### 4.1. Strain Selection and Functional Diversification

The selection of microbial inoculants is the primary determinant of FSBM quality and functional specificity. Industrial applications prioritize GRAS (Generally Recognized as Safe) [59] strains with high enzymatic activity. Fermenting SBM with safe microorganisms such as *Bacillus subtilis*, *Aspergillus oryzae*, *Bifidobacterium lactis*, *Lactobacillus plantarum*, *Saccharomyces cereviseae* has been confirmed to be a cost-effective alternative to improving the functional and nutritional characteristics of SBM [57,60].

#### 4.1.1. Fungal Strains (*Aspergillus oryzae*, *Rhizopus oligosporus*)

Fungal strains are chosen for their higher secretion of extracellular enzymes (cellulases and phytases). Their primary industrial function is the physical disruption of the soybean cell wall matrix and the liberation of chelated minerals. *Pleurotus ostreatus* ACCC 50476 exhibited a greater efficiency in fermenting SBM than *Flammulina velutipes* ACCC 51540 and *Hericium erinaceus* ACCC 50268. The strain was found to enhance the physicochemical properties and nutritional value [46]. Another study selected strains of *Aspergillus awamori* to ferment SBM; after the process, there was an improvement in the replacement level of SBM from 30% to 45% [61].

#### 4.1.2. Bacterial Strains (*Bacillus subtilis*, *Lactobacillus* spp.)

*B. subtilis*, used for fermentation, is preferred for its robust protease production, which breaks down allergenic proteins (glycinin) into bioactive peptides and enhances digestibility and intestinal health in animals. *B. subtilis* is preferred for its robust protease production, which breaks down large allergenic globulins into low-molecular-weight bioactive peptides (<1000 Da); these specific fractions are responsible for enhancing intestinal histomorphogenesis and mucosal immunity [62]. *Lactobacillus* species are selected for rapid acidification, which serves as a bio-preservative against pathogens during storage and transport. A study confirmed that *Lactobacillus acidophilus* LA14-1 reduced the concentration of vibrio bacteria in the intestines and promoted the control of intestinal pathogenic bacteria [63].

#### 4.1.3. Multi-Strain Co-Fermentation

The efficacy of multi-strain co-fermentation is rooted in a dual-stage inoculation logic. In this model, fungal species first catalyze the degradation of recalcitrant structural fibers, thereby liberating simple sugars and increasing the surface area for subsequent bacterial colonization and rapid proteolysis. A study achieved a mixed-strain co-fermentation of SBM with *Bacillus subtilis* N1 and *Lactobacillus plantarum* FPS 2520; the results proved that fermentation drastically reduced total cholesterol, triglycerides, and low-density lipoprotein cholesterol levels in the plasma of mice [64]. Research has revealed that lactic acid bacteria facilitate protein degradation, while lactic acid bacteria with high intestinal adhesion also have antibacterial characteristics [65]. Recently, *Bacillus velezensis*, *Saccharomyces boulardii* and *Enterococcus faecium* were utilized for a one-step mixed culture fermentation of SBM, and the results revealed that based on microbial protease activity, beneficial small peptides significantly increased, which enhanced the palatability of soybean meal and reduced ANFs [66]. This process simplified the operation of mixed fermentation and could be employed in the SSF of feed to improve the beneficial properties of FSBM.

### 4.2. Technical Restrictions and Processing Bottlenecks

Transitioning from laboratory-scale to industrial-scale SBM fermentation presents significant engineering challenges, primarily within SSF systems.

#### 4.2.1. Heat Accumulation

In large-scale beds (over 500 kg), microbial metabolism generates significant metabolic heat. Unlike submerged fermentation, SSF has low water content, making heat dissipation difficult. Enzyme activity can be affected by the temperature of the fermentation system [67]. For instance, the degree of hydrolysis of the proteins fermented with *Bacillus natto* increased when the temperature was raised from 28 °C to 43 °C, resulting in the fermentation rate constantly increasing [67].

#### 4.2.2. Moisture and Oxygen Gradients

Maintaining uniform moisture and oxygen transfer throughout a massive soybean pile is difficult. This often results in clumping and anaerobic pockets where undesirable spoilage organisms (like *Clostridia*) can grow. In solid fermentation, the substrate contains enough moisture to support the growth of microbes without free water compared to liquid fermentation. Solid fermentation can enhance the free radical scavenging (hydroxyl and DPPH), iron chelation property, and significantly reduce the catabolic inhibition of SBM [68,69].

#### 4.2.3. Sterilization Costs

SBM is naturally contaminated with high levels of native bacteria. Industrial-scale autoclaving (steam sterilization) is energy-intensive and expensive [70]. Many producers move toward controlled fermentation where high inoculation doses are used to out-compete native flora, though this carries a higher risk of batch failure.

### 4.3. Economic Viability

#### 4.3.1. Economic Value in Livestock

The economic justification for adopting FSBM over conventional SBM is primarily dictated by improvements in the FCR and the resulting return on investment (ROI). In swine nutrition, particularly during the critical weaning transition, the inclusion of FSBM has been shown to enhance FCR by approximately 5–10% [71]. In large-scale intensive operations, this increased feed efficiency often offsets the processing premium associated with fermentation.

Beyond direct feed efficiency, the economic value of FSBM is augmented by secondary physiological benefits, most notably improved intestinal morphology (increased villus height), and the reduction of ANFs contributes to lower morbidity and mortality rates, thereby reducing the overhead costs associated with therapeutic antibiotic interventions and veterinary care [72]. Consequently, while the marginal cost per ton of FSBM is higher than that of raw SBM, the net economic output, measured by shortened days to market and improved carcass uniformity, positions fermentation as a high-value bioprocessing strategy for modern livestock production.

#### 4.3.2. Bio-Economic Analysis: Profitability of SBM Replacement

The profitability of replacing SBM with FSBM is determined by the net feed efficiency (NFE) rather than the initial purchase price. Industrial estimates suggest that the fermentation and subsequent drying of SBM increase the production cost by approximately 15–25% compared to solvent-extracted SBM. However, this processing premium is mitigated by several performance-based economic offsets:

##### FCR and Production Efficiency Indices

Poultry fed 2.5% FSBM showed a significant body weight gain and a decreased FCR [12]. Recent data indicate that FSBM significantly improves the European Production Efficiency Index (EPEI) in poultry by enhancing nutrient utilization and reducing waste [73]. In finishing pigs, even marginal reductions in FCR (0.16%) contribute to a substantial lowering of animal production expenses over a full growth cycle [74].

##### Comparative Net Profitability and Margin Analysis

The economic efficiency of FSBM is further supported by its role as a high-value substitute in diets where traditional ingredient prices are volatile. Studies utilizing FSBM have reported the highest net profit and profitability ratios when compared to unfermented diets [75]. For instance, the marginal inclusion of just 2% FSBM has been shown to positively influence growth performance while remaining economically superior to unfermented controls [76].

##### Resource Optimization

Fermentation serves as a cost-effective method to improve the nutritional density of feed ingredients, facilitating a “low-cost economical diet” that compensates for the rising prices of traditional meal [77]. By boosting the digestion of low-quality protein fractions and lowering nutrient waste through exogenous enzyme activity, FSBM improves the overall return on investment (ROI) for intensive livestock operations [73].

##### Mortality and Health Economics

The replacement of SBM with 5% FSBM has been linked to improved immunity, reduced mortality in finishing pigs [78] and decreased therapeutic antibiotic expenditures. In high-density operations, the economic value of an animal from initial birth and early-stage feeding contributes substantially to the overall profitability of the FSBM-based diet [74]. The profitability effectively neutralizes the higher ingredient cost, yielding a net positive ROI through reduced death and lower veterinary costs, as summarized in Table 2, where the physiological gains in FCR and growth are translated into quantifiable economic indicators.

## 5. Soybean Meal Fermentation Mechanism: Its Influence on Microbial Ecology, Physicochemical, and Functional Properties

Fermentation can enhance SBM’s nutritional value, which involves improving the amino acid profile, biodegradation, and reduction of oligosaccharides and phytic acid [14,79]. Recent studies have increasingly focused on how fermentation alters the protein profile of soybeans [80]. The primary biochemical transformations during fermentation are driven by protein hydrolysis, facilitated by protease enzymes, which are secreted by various microorganisms [81]. Zheng’s study evaluated the major ANFs in SBM and the effects of fermentation on soybean meal protein (SBMP) microstructure using *Bacillus* both before and after fermentation. Their findings showed that *in vitro* absorbability and digestibility increased by 18.9% and 8.7%, respectively, while the FSBM showed better properties than SBM, and the ANFs such as β-conglycinin, glycinin, and TI decreased by 70.3%, 86.0%, and 95.01% after 24 h fermentation [37].

Fermentation also causes the microstructural breakdown of SBM proteins, altering their nutritional and functional properties. Liu’s group observed that the surface hydrophobicity of soybean protein isolate negatively correlates with α-helix structures and positively correlates with β-sheet and random coil structures [82]. Fourier-transform infrared spectroscope (FTIR) and scanning electron microscope (SEM) were employed in another study to analyze the microstructure and protein morphology of FSBM. It was found that fermentation could enhance the nutritional quality of FSBM, modifying the β-sheet structure and disrupting the original SBM structure [37]. In summary, FSBM peptides, especially β-conglycinin and glycinin, are either released by fermentation microbes or derived from protein hydrolysis caused by biochemical changes and enzymatic activities in microbes. Bioactive peptides have metabolic and functional properties, such as antimicrobial, immune-modulating, and antioxidant effects, and offer health benefits depending on the composition and biochemical transformations of amino acids [36].

Substrate fermentation occurs under specific moisture, redox, and temperature conditions, hence promoting the growth of microorganisms, which results in the biotransformation of various substrates at different rates. Additionally, in FSBM, ethanol, lactic acid, formic acid, and acetic acid are the main low-molecular-weight fermentation products. These metabolic products, along with the microbes themselves, can affect the gut microbiome in animals. The influence depends on factors such as fermentation technique, FSBM dosage, substrate properties, and microbial strain [83,84]. Moreover, as nutrients decrease and metabolites build up during the stationary phase of fermentation, the production of specific organic compounds slows, leading to increased levels of soluble proteins and amino acids [85].

Fermentation enhances the quality of SBM, as it affects its functional properties. These properties are associated with the protein surface and structure, rheological characteristics, and hydration [86]. They are crucial in determining water-holding capacity, thermal stability, emulsifying properties, consistency, and gel-forming ability of SBM. In addition, SBM’s complex proteins and peptides have significant biological activities, such as emulsifying and stabilizing substances [57]. In the feed industry, enhancing the emulsion capacity of feed is key, as it boosts livestock growth performance and increases feed stability by improving the digestibility of fatty acids [87]. Lu’s work indicated that the natural fermentation of SBM increases emulsifying stability and the activity index. It was observed that fermentation loosens the protein structure of SBM, exposing hydrophobic deposits that can interact with hydrophobic molecules, such as oils. These functional improvements in animals can impact their health directly or indirectly, affecting aspects such as fermentation in both the foregut and hindgut, gut health, and bowel movement [60].

**Figure 2:**
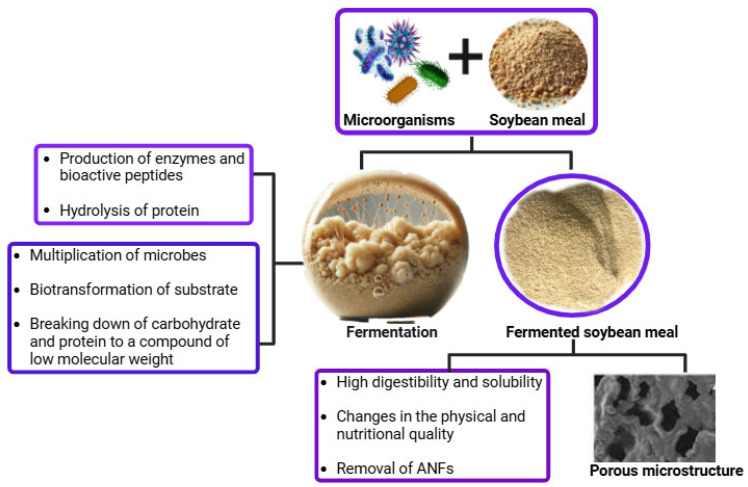
Pictorial illustration of the fermentation mechanism of soybean meal to enhance its nutritive value.

Overall, breaking down proteins into numerous small peptides and eliminating various anti-nutritional factors, which act as carriers for beneficial digestive enzymes, antibiotics, and bacteria, enhances the nutritional value of SBM after fermentation (Figure 2). Additionally, fermentation generates multiple mycoproteins and bioactive compounds. During this process, microorganisms convert complex, large-molecular organic compounds into simpler molecules in the feed that can be effortlessly absorbed by animals. This process also involves the production of various beneficial bacterial proteins and metabolic compounds. During fermentation, when microbes consume organic compounds, it increases the CP content. This results in the protein’s “concentration effect” and a sour flavor that can arouse the appetite and improve animal feed ingestion. Fermentation enhances the solubility and emulsification of proteins, which is significant for emulsion stabilization [88].

## 6. Technical Bottlenecks: Safety Risks and Microbial Stability

Fermentation is a robust bioprocessing tool, and its industrial application is constrained by inherent risks related to metabolite safety and the maintenance of microbial viability.

### 6.1. Mycotoxin Contamination and Secondary Metabolites

A significant bottleneck in fungal fermentation, particularly when utilizing *Aspergillus* or *Rhizopus* strains, is the potential for mycotoxin synthesis [89]. Although industrial starter cultures are selected for their non-toxigenic profiles, the high-moisture and warm-temperature conditions required for optimal fermentation (e.g., 26–28 °C and moisture content higher than 18%) create a biological trade-off, as these parameters also favor the growth of opportunistic contaminants such as *Aspergillus flavus* [90]. Consequently, improper environmental control during large-scale SSF can trigger the production of the most common mycotoxins present in fermented SBM, aflatoxins (AFs). AFs are of great concern due to their severe harmful effects on animal health [91]. They can induce teratogenic, carcinogenic, hepatotoxic, and mutagenic effects when consumed or absorbed through the skin, even at minute concentrations [92]. To mitigate these risks, the industry increasingly relies on high-dose inoculation of competitive starter cultures and rigorous good manufacturing practices (GMP) to ensure that the final FSBM remains within regulatory safety limits.

### 6.2. Probiotic Viability and Post-Processing Stability

The efficacy of bacterial fermentation is often limited by the instability of the probiotic populations. A critical viability gap occurs during the mandatory post-fermentation drying phase, where high thermal loads required to reduce moisture for storage can significantly diminish live microbial counts (e.g., *Lactobacillus* spp.) [79]. Furthermore, the probiotic effect is subject to chronological degradation; studies indicate that viable cell counts can decline during storage periods. This instability complicates the standardization of minimum effective doses in animal diets. Current research is therefore shifting toward the use of protective encapsulation or the focus on postbiotics, the heat-stable metabolic byproducts, which may retain functional benefits even if live cell counts decrease during processing [93].

## 7. Application of Fermented Soybean Meal in Animal Production

Animal production is increasingly using FSBM as a high-quality protein source. The fermentation process enhances SBM by reducing ANFs like TIs and indigestible oligosaccharides, which can hinder nutrient absorption. Fermentation also increases the availability of essential amino acids, bioactive peptides, and vitamins. As a result, animals fed FSBM often exhibit improved growth performance, better gut health, and enhanced immune responses [94]. FSBM has been successfully incorporated into the diets of various livestock, including pigs, poultry, ruminants, and aquatic species, leading to more efficient feed utilization and overall productivity in animal farming.

### 7.1. Application of Fermented SBM in Ruminant Diets

Researchers found that FSBM enhanced both milk yield and milk protein content in lactating cows [95]. FSBM-fed calves not only experienced reduced weaning stress, marked by lower levels of pro-inflammatory mediators, but also showed improved growth performance compared to calves fed SBM during cold weather [96]. Calves challenged with lipopolysaccharide (LPS) were fed FSBM, and it was found that it could alleviate weaning stress and enhance their immune status. Higher levels of haptoglobin, LPS-specific IgA, and LPS-specific IgG were observed after FSBM feeding, while cortisol levels in the blood were reduced [16].

**Figure 3:**
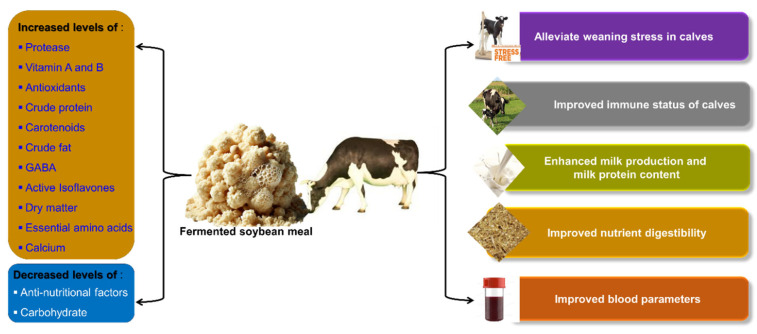
Diagrammatic illustration of the benefits of FSBM consumption in cows and calves.

A published work reported that FSBM could serve as an effective calf starter for enhancing growth and health in weaned calves by mitigating the weaning stress response. This effect was attributed to the presence of various functional molecules and their upgraded nutritional value. FSBM reduced stress by lowering levels of acute-phase protein stimulation and pro-inflammatory cytokines, leading to better feed intake, growth and health in pre-weaned calves [96]. Recently, Holstein cows fed FSBM had increased levels of milk protein yield, milk urea nitrogen (MUN), milk fat yield, and fat-corrected milk (FCM), but there was a decrease in milk somatic cell count (SCC). The population of Spirochaetota and Fibrobacterota phyla increased, while Pseudomonadota decreased [95]. Another study revealed that replacing SBM with FSBM can enhance calf performance by influencing rumen fermentation and altering the ruminal bacterial community [97]. Furthermore, the amount of rumen undegraded protein (RUP) in FSBM may increase as a result of intensive heat treatment (cooking) applied before fermentation. Protein fractions like RUP are known to influence calf performance and digestibility [98]. Figure 3 summarizes the benefits of FSBM for pigs, and Table 3 outlines applications of FSBM to ruminants.

Despite its potential benefits, there is surprisingly inadequate data on the use of FSBM in the diets of adult ruminants. Most research has focused on young calves, examining how FSBM affects their immune responses and productivity. To the best of our knowledge, the only study involving adult ruminants focused on lactating cows, and it was observed that FSBM influenced the rumen’s bacterial microbiota and improved rumen fermentation processes [99]. Currently, there are no studies on the utilization of FSBM in goats and sheep, leaving a gap in understanding how adult ruminants respond to FSBM. One possible reason for this gap is that fermented feeds might present challenges in adult ruminants due to the complexity of their digestive systems and mechanisms of processing feed, which relatively differ from monogastric animals. Additionally, younger ruminants have their gastrointestinal tracts more analogous to those of monogastric animals. Furthermore, unlike young ruminants, adult ruminants are less dependent on the amino acid profile of vegetable proteins. Their extensive gut microflora can change nitrogenous compounds into high-value rumen microbial CP, making the specific amino acid composition of their feed less essential.

### 7.2. Application of FSBM in Pig Diets

Research has explored the use of FSBM in monogastric animals, particularly pigs, and has provided valuable insights (Table 4). For instance, numerous researchers have demonstrated a link between changes in the chemical composition of FSBM and the enhanced digestibility of amino acids and CP. A notable adjustment is the removal of ANFs that are naturally present in SBM. ANFs are generally detrimental to animal growth because they reduce feed intake, hinder nutrient digestion, lead to metabolic disorders, disrupt gastrointestinal function, and can cause stress [46,51,100]. Weaned piglets that were fed FSBM showed a reduction of approximately 60–96% in the levels of TIs, β-conglycinin, and glycinin. Additionally, there was an increase in digestible nutrients, as fermenting microorganisms could break down macromolecules, enhancing the absorption and digestion of amino acids and CP [7]. Feeding weaned piglets 6% FSBM led to increased growth performance because of the reduced immunological challenge and better nutritional status of the diet [101].

**Figure 4:**
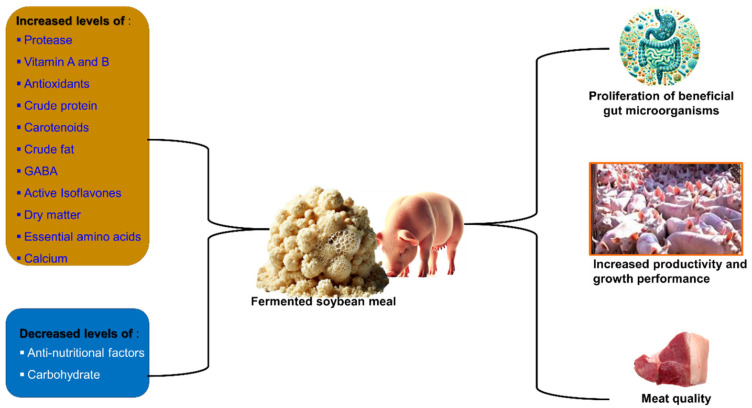
Diagrammatic illustration of the consumption benefits of FSBM in pigs.

FSBM elevated the levels of soy isoflavones (SIFs) in the form of aglycones, thereby reducing oxidative stress in sows. Substituting SBM with FSBM in the diet of gestational and lactating sows helped alleviate oxidative stress, enhance milk composition, and significantly boost the average daily growth of suckling piglets after birth [104,109]. Recently, a study was conducted on mid- and late-gestation sows and provided a basis for the rational addition of FSBM to the sow diet. It showed that the metabolizable energy (ME) and digestible energy (DE) values for mid- and late-gestation of FSBM were 17.70 MJ/kg; 18.22 MJ/kg, and 16.81 MJ/kg; and 17.81 MJ/kg, respectively [110]. Finishing pigs fed FSBM showed an increase in serum glucagon levels; glucagon, an insulin-counteracting hormone, is essential for stimulating glucose production and its release from the liver [111]. Another study confirmed the beneficial application of FSBM in pig farming; the results revealed that FSBM enhanced the meat quality and growth performance of finishing pigs. This could be attributed to the increased muscle oxidation capacity, serum, and nutrient digestion [102]. In finishing pigs, FSBM not only enhanced the meat quality and growth performance but also controlled the intestinal diversity of the microbial population. It increased the abundance of Bacteroidia, Bacteroidetes, Bacteroidales, and Prevotellaceae [74]. Figure 4 outlines the applications of FSBM to ruminants.

Several studies have demonstrated the positive impact of FSBM on pig production parameters [17,102,112,113,114]. These findings align with earlier research showing that FSBM products enhanced pig production metrics by improving the digestibility of essential nutrients [115]. The improved digestibility in pigs is likely due to the enzymatic activities produced by probiotics [116]. In the production of piglets, soybean protein concentrate (SBPC) and plasma protein (PP) are the most available protein sources for enhancing the immunity, production performance, and overall health of the animal. However, these protein sources are expensive and limit their application in the swine diet. Therefore, Yuan’s group substituted high-quality, low-cost FSBM for SBPC and PP to improve piglet performance. Their results showed that FSBM had a greater FCR and average daily gain (ADG), which improved the diet, while diarrhea and mortality rates decreased remarkably [17].

FSBM transformed the composition of microbiota and metabolites in the large intestine of piglets and improved their growth performance [103,117,118]. Eighty piglets were used to demonstrate the effectiveness of FSBM as a protein source for growth. The study concluded that FSBM can be used as a substitute for SBM. At day 7, 21 and 42, a significant increase in body weight was observed [112]. Recently, FSBM decreased anti-nutritional factors such as phytate phosphorus and neutral detergent fiber by 59.2% and 27%, respectively. In addition, there was a 5% increase in crude protein and a successful breakdown of large antigenic proteins. Crude protein had a higher digestibility in piglets fed FSBM during the third and fourth weeks. In week 4, the gain-to-feed ratio experienced significant improvements [119]. Existing research suggests that adding FSBM to the diet can help maintain health and boost economic efficiency. However, when formulating swine diets, it is important to account for the differences in available energy among various FSBM samples [25].

### 7.3. Application of FSBM in Poultry Diets

#### 7.3.1. Broilers

Studies have documented the effects of fermented SBM and other fermented feeds on meat quality, production performance, physiological activities, and egg quality of poultry (Table 5). For instance, FSBM promoted FCR and ADG during the growth and whole phases of broilers. There was a significant improvement in serum immunoglobulin concentrations. Substituting FSBM in the diet of broilers altered the cecal microbial community toward a healthier composition by increasing beneficial bacteria and decreasing potentially harmful ones [120]. These findings suggest that FSBM could serve as a novel feed resource to enhance growth performance and regulate intestinal microbiota in animals [15]. After partial replacement of SBM with FSBM, there was a significant increase in peptide, crude protein, and total phenolic contents, while the activity of TI decreased by 74% compared to SBM [121].

**Figure 5:**
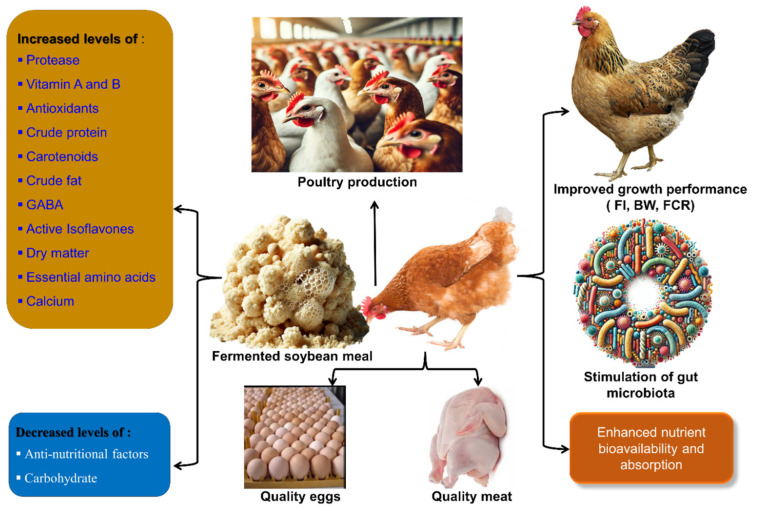
Diagrammatic illustration of the consumption benefits of FSBM in poultry.

FSBM has been proven to enhance growth performance and improve gut health in broiler chickens (Figure 5). However, due to its high cost, FSBM is typically used only in pre-starter diets. A study highlighted that partial replacement of SBM in broiler diets not only enhances meat quality but also extends its shelf life and boosts consumer appeal. While these findings are promising, further research is essential to fully uncover the benefits of substituting SBM with FSBM in improving both the growth and meat quality of broiler chickens [125]. Functional ingredients present in fermented feeds can be considered an effective approach to reducing the colonization of gut pathogens in broiler chicks [129]. It was reported that young broiler chicks fed FSBM had improved growth performance and intestinal characteristics, lowered *Salmonella* colonization, and enhanced immune response to *Salmonella typhimurium* infection in young broiler chicks [130]. Tsai et al. [124] demonstrated that the two-stage FSBM process boosts immune function and strengthens tight junctions in the jejunum of broilers, thereby promoting overall health and improved performance. Incorporating double-fermented soybean meal (DFSBM) into broiler diets enhanced feed efficiency, nutrient digestibility, and amino acid transport, leading to improved weight gain and better muscle nutritional value in the birds [14]. Incorporating 3% or 6% FSBM into chicken diets unlocked impressive benefits, boosting total antioxidant potential (fraP) and plasma glutathione levels. Chickens fed FSBM also showed higher total protein and HDL cholesterol levels, enhanced aspartate aminotransferase activity, and lower urea content.

Beyond these positive impacts on protein, lipid metabolism, and antioxidant defenses, a 6% FSBM diet significantly improved body weight gain. In short, replacing FSM with a 6% share of FSBM proves to be a smarter and more effective choice for optimizing chicken health and growth [127]. The addition of 10% FSBM to broiler diets delivered impressive results, significantly reducing *IL-4* mRNA expression in key immune organs like the spleen and bursa of fabricius. Broilers fed this enhanced diet also exhibited notably lower levels of serum anti-soybean IgG. These findings reveal that the inclusion of *B*. *subtilis* and protease during the SSF process of SBM effectively reduces ANFs. Even more exciting, the 10% FSBM diet appears to actively suppress allergic immune responses in broilers, offering a promising dietary strategy for improved health [18].

#### 7.3.2. Laying Hens

FSBM has exhibited probiotic benefits, and when fed to Japanese quails, it not only boosts growth performance but also cultivates a healthy balance of beneficial gut microbiota in the crop and ceca. It enhances the small intestinal structure and optimizes the serum lipid profile, delivering results comparable to a powerful probiotic supplement [45]. The nutritional quality of feed for laying hens was carefully analyzed before and after fermentation, and four levels of fermented feed were introduced to replace unfermented feed, aiming to explore its impact on the gut health of laying hens during their peak production period. The results are promising: fermented feed not only enhances the intestinal structure and strengthens barrier functions but may also aid in reshaping the cecal microbiome, supporting overall gut health in laying hens [131,132]. A recent study has shown that incorporating 2.5% or 5.0% FSBM into the diets of laying hens enhances the digestion of amino acids, gut health, immune activities, egg production, and quality. Additionally, further analysis revealed that FSBM positively influenced the gut microbiota, promoting the growth of beneficial microbes [123]. Turkey hens fed 9% and 10% FSBM showed enhanced growth and a positive impact on the histology of the small intestine [122]. Additionally, in another study on chickens, a dosage of 6% of FSBM improved the intestinal morphology (shown by increased crypt depth and villi length). It also resulted in the decline in the overall quantity of coliforms and fungi in the jejunum [128].

## 8. Challenges and Opportunities of Fermented Soybean Meal

SBM has been extensively studied and used as a feed source for livestock over the years. However, FSBM is increasingly being recognized as an alternative plant protein for ruminants, pigs, and poultry. Despite the clear benefits FSBM provides in livestock diets, there are several areas that require further research.

The literature reported has primarily focused on using microorganisms from the fungal kingdom, such as *Candida* and *Aspergillus* species, and Gram-positive bacteria like *Bacillus* spp., for fermenting SBM. While these microbial inoculants are commonly used, research on the application of *Lactobacillus* spp., which offer notable probiotic benefits, remains limited. With current restrictions on animal growth promoters in feed, it is important to investigate other microbial strains, individually or in combination, that can boost SBM’s protein content, break down ANFs, and enhance gut health by improving digestion and nutrient absorption in the gastrointestinal tract.

Secondly, FSBM is a lipid-rich feed component, containing unsaturated fats that may alter the fatty acid profile of animals [133]. However, there is a paucity of data on its specific effects on the digestive functions of animals, particularly in relation to lipid and protein metabolism. Further studies are necessary to investigate gene expression related to these metabolic pathways. Additionally, fermentation breaks down large protein molecules into smaller peptides that are readily absorbed, which may accelerate digestive system development in young animals such as calves, piglets, and chicks. Therefore, studies on cytology and genetics are crucial to understanding how FSBM impacts intestinal growth and nutrient absorption in young animals, potentially improving their health and growth rates.

Another key factor identified in the literature is the variation in fermentation time used in different studies, ranging from 12 h to 7 days. The duration of fermentation influences the composition of FSBM, particularly in terms of protein content and the production of non-protein nitrogen fractions, such as small peptides and free amino acids. Research has indicated that these fractions increase with longer fermentation times. However, further investigation is required to identify microbial strains that can reduce fermentation times while maintaining or improving production efficiency, which could ultimately reduce the cost of production.

Moreover, competition for soybeans across food industries, animal feed, and biofuel production has driven up costs. According to USDA reports, the high cost of SBM remains a significant challenge for the poultry and pig industries, making the potential replacement of SBM with FSBM a promising solution for improving feed efficiency [14]. However, few reports have assessed the cost-effectiveness of formulating and using FSBM compared to the possible benefits it provides. Imminent research should include comprehensive cost analyses to evaluate the economic feasibility of partially or fully replacing SBM with FSBM, especially in large-scale commercial operations.

Additionally, the majority of the soybeans cultivated globally are genetically modified (GM), which has raised public concerns. Although numerous studies have reported no significant adverse effects of GM feed on livestock or their products, some research has highlighted potential health risks in humans. It is not yet clear whether fermentation could alleviate these concerns; further investigation is required to explore the potential of FSBM in addressing this issue.

Finally, the environmental implications of feeding livestock FSBM, especially in the long term, must be considered. Researchers have proven that diet composition can alter the production of enteric methane via the manipulation of microbes in the rumen. Research has indicated that fermented feeds like FSBM can enhance lactic acid bacteria populations, which in turn may reduce methane emissions by creating a more favorable gut environment. Additionally, microbes involved in FSBM fermentation produce enzymes that improve fiber digestion and lower methane output. However, certain compounds like saponins and tannins, while having methane-reducing potential, may pose health risks at higher concentrations. FSBM, with its minimal anti-nutrient content, could offer a safer and more effective alternative for reducing methane emissions without harming animal health. In adult ruminants, the role of FSBM and its ability to modulate the rumen network and mitigate emissions of methane requires further research. Overall, while FSBM holds great promise as a sustainable, nutritious feed alternative, further research is required to unlock its full potential in minimizing environmental impacts, reducing costs, and improving livestock health.

### 8.1. Economic Trade-Offs and Industrial Return on Investment

A primary challenge for the widespread adoption of FSBM in commercial animal husbandry remains the initial processing premium, which typically increases the cost per ton. However, the emerging opportunity lies in the bio-economic offset provided by enhanced animal health and production velocity. In intensive systems, the profitability of FSBM is realized through a dual mechanism:

#### 8.1.1. Production Gains

The documented 5–10% improvement in FCR and the reduction in days to market significantly lowers the total feed volume required per growth cycle, effectively neutralizing the higher ingredient cost within the total ration.

#### 8.1.2. Health Economics

FSBM acts as a preventive health tool, where the reduction in enteric disorders and weaning-associated mortality lowers the overhead of veterinary interventions and antibiotic use.

Future industrial success will likely depend on refining SSF kinetics to further reduce energy costs (e.g., drying), thereby widening the profit margin and making FSBM an essential cost-offsetting strategy in post-antibiotic livestock production.

## 9. Conclusions

A high-quality protein diet providing optimal amino acid profiles is a critical requirement in animal feed formulations to ensure safety and economic efficiency. FSBM has demonstrated significant potential as a protein source capable of partially or entirely replacing conventional SBM by effectively mitigating allergenic ANFs, such as phytates, TIs, saponins, and tannins. Fermentation with targeted microbial inoculants facilitates the production of bioactive compounds, including antimicrobial, antioxidant, and immune stimulatory metabolites, while simultaneously altering the SBM microstructure to enhance protein solubility and nutrient bioavailability. However, the reported functional benefits, including potential probiotic effects, must be interpreted with caution; large-scale meta-analyses are required to validate the consistency of these outcomes across varied commercial settings. Future industrial adoption remains contingent upon the standardization of microbial strains and the rigorous optimization of fermentation conditions, particularly moisture and thermal kinetics. While FSBM exhibits documented improvements in gut morphology and growth performance, further research must address the synergy between multi-strain inoculants and the long-term environmental implications of production to ensure sustainable and scalable implementation. Ultimately, the question of industrial profitability is resolved through a bio-economic offset model. While the fermentation process introduces an initial processing premium, the documented 5–10% improvement in FCR and the significant reduction in morbidity-associated costs render the investment highly profitable in intensive animal husbandry. By maximizing nutrient extraction and minimizing veterinary overhead, FSBM serves as a self-compensating investment that enhances both the biological performance and the net profit margins of modern livestock enterprises.

## Figures and Tables

**Table 1:** The nutritional density of SBM constituents.

Nutrient Category	Typical Content (% DM)	Principal Nutritional Role/Impact
Crude protein	44–48%	High lysine (91%); benchmark for AA balance
Total lipids	2–17%	High metabolic energy; source of tocopherols (Vit. E)
Carbohydrates	35–40%	Includes NSPs and flatulence-inducing oligosaccharides
Minerals	4.5–6.4%	Primarily K, P, and Mg; P is largely phytate-bound
Antigens	Variable	Glycinin and conglycinin; induce intestinal inflammation
Protein inhibitors	variable	Kunitz/Bowman-Birk; induce pancreatic hypertrophy

**Table 2:** Bio-economic performance indicators and profitability metrics of fermented soybean meal (FSBM) versus conventional soybean meal (SBM) in intensive livestock and poultry production.

Parameter	Raw SBM (Control)	Fermented SBM	Economic Impact	References
Feed Conversion Ratio (FCR)	Baseline	Significant increase	Primary Profit Driver: 5–10% reduction in total feed volume required.	[71]
Production Index (EPEI)	Baseline	Significant increase	Efficiency: Higher net income per broiler crop.	[73]
Net Profit Ratio	Baseline	2%	Return on Investment: Highest profitability ratios at 2% inclusion	[76]
Mortality/Morbidity	Baseline	5%	Cost Mitigation: Lower veterinary fees and fewer “lost” animals.	[78]

**Table 3:** Application of fermented soybean meal (FSBM) in ruminant production and health.

Physiological Status of the Animal	Fermentation Microbe(s)	Dose of Inoculant	Level of Inclusion (%)	Advantages	Reference
Calves	*Bacillus subtilis*	10,000 cfu/g of SBM	9–13.5% FSBM inclusion replaced 33–50% SBM	• Performance: ↑ Starter intake and growth • Microbiome: Altered rumen bacterial abundance and metabolites	[97]
Lactating dairy cows	*Lactobacillus* spp., *Bacillus subtilis*, and *Saccharomyces cerevisae*	NS	Total replacement (5.55% FSBM inclusion)	• Rumen Function: ↑ Propionate and valerate levels; ↑ Rumen pH • Microbiome: Enriched *Saccharofermentans* and *Fibrobacter* spp.	[99]
Cows	NS	NS	Total replacement	• Lactation: ↑ Milk protein, fat yield, and FCM • Metabolism: ↓ Somatic cell count; ↑ AA biosynthesis genes	[95]
Weaned calves	*Bacillus subtilis*	10,000 cfu/g of SBM	9–13.5% FSBM inclusion replaced 33–50% SBM	• Stress: Mitigation of weaning stress via ↓ proinflammatory mediators • Performance: Optimized growth performance	[96]

FSBM—fermented soybean meal; SBM—soybean meal; NS—not stated; AA—amino acid; FCM—fat-corrected milk; ↑—increased; ↓—decreased.

**Table 4:** Application of fermented soybean meal (FSBM) in pig production and health.

Physiological Status of the Animal	Fermentation Microbe(s)	Dose of Inoculant	Level of Inclusion (%)	Advantages	Reference
Nursery pigs	*Bacillus subtilis* CP-9	NS	34% FSBM inclusion (as-fed basis) as the only dietary protein source	• Digestibility: ↑AID of Ash, ADF, NDF, CP, and DM	[62]
Finishing pigs	*Aspergillus oryzae* GB-107	NS	8% replacement	• Performance: ↑ Meat quality and growth • Microbiome: ↑ *Bacteroidetes* and *Prevotellaceae* populations	[74]
Finishing pigs	*Bacillus subtilis*	1 × 10^8^ cfu/g	50% replacement	• Performance: ↑ Growth and carcass traits; ↑ Meat quality. • Metabolism: ↑ Muscle antioxidant capacity; altered gene expression in *longissimus thoracis*	[102]
Piglets	*Bacillus subtilis* BS12	10^7^ to 10^8^ cfu/mL	10% inclusion	• Physiology: ↑ Tight junction protein/mucin expression • Health: ↓ Serum IL-1β, IL-6, and D-lactate; ↓ ileal macrophage infiltration	[103]
Piglets	*Lactobacillus casei*, *Bacillus subtilis*, and *Hansenula anomala*	1 × 10^6^ cfu/g	3.75–7.5% substitution for SBM and wheat bran	• Performance: ↑ ADG; ↓ FCR • Microbiome: ↑ LAB counts; ↓ *E. coli* counts; ↑ fecal enzyme activity	[17]
Sows and piglets	*Lactobacillus reuteri* and *Aspergillus oryzae*	NS	2–4% inclusion replaced 50% SBM	• Health: ↓ Oxidative stress (↑ SOD; ↓ MDA/Cortisol); ↑ Colostrum IgG • Reproduction: ↑ Serum estrogen and growth factors	[104]
Piglets	*S. cerevisiae* JM 102, *Bacillus lactis* RG 103, and *Bacillus subtilis* KC 101	1 × 10^9^ 2.5 × 10^9^ 1 × 10^10^ cfu/g	5% dry FSBM and 7.33% wet FSBM inclusion	• Metabolism: ↑ Carbohydrate metabolites; ↓ Protein metabolites in large intestine • Performance: Improved growth performance	[103]
Weaned pigs	*Bacillus subtilis*, *Saccharomyces cerevisiae*, and *Lactobacillus plantarum*	10^8^ cfu/g	10% replacement	• Morphology: ↑ Villus height (duodenum/ileum); ↓ Crypt depth • Immunity: ↑ Serum IgA, IgG, and IgM; ↑ Total serum protein	[105]
Weaned pigs	Yeast, *Bacillus subtilis*, and *Lactobacillus*	NS	Total replacement (32% inclusion on an SS-fed basis)	• Efficiency: ↑ AID of AA, CP, and energy • Health: ↑ Antioxidant capacity and intestinal integrity	[7]
Weaned piglets	*S. cerevisiae*, *Streptococcus thermophilus*, and *B. subtilis* MA139	NS	6% replacement	• Performance: Improved ADFI and ADG • Health: Mitigated weaning-associated immunological challenges	[101]
Weaned piglet	*Escherichia faecium* SLB120	1 × 10^8^ cfu/g	Total replacement (39% inclusion of FSBM on an as-fed basis)	• Digestibility: ↑ AID of ME, DM, CP, and Nitrogen	[55]
Weaned piglets	*S. cerevisiae*, *Streptococcus thermophilus*, and *Bacillus subtilis* MA139	1 × 10^7^ cfu/g	3–6% replacement (on an as-fed basis)	• Performance: Improved ADFI and ADG • Health: Mitigated weaning-associated immunological challenges	[101]
Weaned piglets	*Clostridium butyricum*, *Lactobacillus acidophilus*, *Lactobacillus salivarius*, and *Lactobacillus delbrueckii*	1 × 10^8^ cfu/g	5% inclusion	• Health: ↓ Incidence of diarrhea; ↑ Serum IgG and IgA • Microbiome: ↑ Fecal LAB; ↓ Enterobacteriaceae populations	[106]
Enterotoxigenic *E*. *coli* challenged piglets	*Bacillus subtilis*, *S. cerevisiae*, and *S. thermophilus*	Mixed at a ratio of 1:1:1	25% replacement	• Health: ↓ Diarrhea; ↓ Pro-inflammatory cytokines; ↓ Mucosal apoptosis • Gut Health: Improved ileal barrier function and growth performance.	[107]
Enterotoxigenic *E*. *coli* challenged piglets	*Bacillus subtilis*, *S. cerevisiae*, and *S. thermophilus*	1 × 10^7^ cfu/mL	6% replacement	• Performance: ↑ Growth efficiency • Microbiome and Immunity: Modulation of cecal microbial diversity and downregulation of pro-inflammatory cytokine gene expression	[108]

FSBM—fermented soybean meal; SBM—soybean meal; NS—not stated; AID—apparent ileal digestibility; AA—amino acid; ADG—average daily gain; ADFI—average daily feed intake; CP—crude protein; DM—dry matter; ME—metabolizable energy; IgA—immunoglobulin A; IgG—immunoglobulin G; IgM—immunoglobulin M; ADF—acid detergent fiber; IL-1β—interleukin 1β; IL-6—interleukin 6; FCR—feed conversion ratio; SOD—superoxide dismutase; LAB—lactic acid bacteria; NDF—neutral detergent fiber; ↑—increased; **↓**—decreased.

**Table 5:** Application of fermented soybean meal (FSBM) in poultry production and health.

Physiological Status of Bird	Fermentation Microbe(s)	Dose of Inoculant	Level of Inclusion (%)	Advantages	Reference
Quails	*Lactobacillus plantarum*, *Bacillus subtilis*, and *Aspergillus oryzae*	10^5^ cfu/mL 10^6^ spores/mL	Total replacement (37% FSBM inclusion)	• Performance: ↑ Weight gain; ↓ FCR • Morphology: Improved small intestine structure • Microbiome: Optimized crop and cecal microbial balance.	[45]
Turkey	*Lactobacillus plantarum*	NS	9% and 10% inclusion	• Performance: ↑ Weight gain • Morphology: ↑ Villus height and VH:CD ratio • Composition: ↓ ANFs; enriched probiotic LAB cultures	[122]
Laying hens	NS	NS	2.5% and 5% inclusion of FSBM	• Production: ↑ Egg quality and production • Physiology: ↑ Apparent fecal AA digestibility; ↑ Antioxidant capacity • Composition: ↑ Mineral and CP density	[123]
Broiler	*S. cerevisiae*, *Bacillus amyloliquefaciens*, and *Lactobacillus acidophilus*	NS	25% replacement of SBM	• Performance: ↑ ADG; ↓ FCR • Immunity: ↑ Serum IgA, IgG, and IgM levels • Microbiome: Positive modulation of cecal microbial diversity	[15]
Broiler	*Bacillus stearothermophilus*	NS	Up to 39% and 42% replacement of SBM in starter and grower diet	• Morphology: Enhanced gut flora and intestinal traits • Immunity: ↑ Thymus and Bursa of Fabricius weights • Microbiome: ↓ *E. coli* populations in duodenum/cecum	[121]
Broiler	*Bacillus velezensis* and *Lactobacillus brevis*	NS	6% FSBM inclusion replaced 17% and 21% SBM in the starter and finisher diets, respectively	• Barrier Function: ↑ *MUC2* and tight-junction gene expression • Immunity: Regulation of jejunal inflammatory factors • Health: Improved intestinal repair during stress.	[124]
Broiler	Yeast, *Bacillus subtilis*, and *Lactobacillus* spp.	NS	2.5%, 5.0%, and 7.5% partial replacement of SBM	• Quality: ↑ Meat quality and nutritional composition • Physiology: Enhanced antioxidant properties.	[125]
Broiler	1st stage: *Asperigillus oryzae* SS_RS-SH (MN894021.1) 2nd stage: *Bacillus subtilis* SB102	10^8^ cfu/mL 10^8^ cfu/mL	50% and 100% replacement of SBM	• Performance: ↑ Growth; ↑ Breast meat nutritional value • Immunity: ↑ Humoral immune response; ↓ Coliform counts.	[14]
Broiler	*Bacillus subtilis*	NS	5% and 10% inclusion of FSBM	• Health: Suppression of allergic immune responses • Composition: Significant reduction in residual ANFs	[18]
Broiler	*Bacillus subtilis*, *Aspergillus oryzae*, *Lactobacillus plantarum*, and *Lactobacillus acidophilus*	10^8^ cfu/mL 10^6^ spores/mL	Total replacement (32%, 34%, and 37% inclusion of FSBM in starter, grower, and finisher diet)	• Metabolism: ↑ Hepatic *IGF-1* expression; ↓ Plasma 3-methylhistidine • Digestibility: ↑ Ileal CP and energy digestibility; ↑ Amylase/protease activity • Performance: ↑ BWG and feed efficiency	[126]
Chicken	*Lactobacillus* bacteria	NS	3–6% replacement of SBM	• Performance: ↑ Weight gain • Blood Profile: ↑ Total protein and HDL cholesterol; ↑ AST activity	[127]
Chickens	*Lactobacillus*	About 10^6^ cfu/g	3–6% FSBM inclusion replaced up to 17–27% SBM	• Morphology: Improved intestinal traits and dressing percentage • Microbiome: ↓ Jejunal fungi and coliform populations	[128]

FSBM—fermented soybean meal; SBM—soybean meal; NS—not stated; VH:CD—villus height to crypt depth; ANFs—anti-nutritional factors; LAB—lactic acid bacteria; AA—amino acid; ADG—average daily gain; ADFI—average daily feed intake; CP—crude protein; BWG—body weight gain; IgA—immunoglobulin A; IgG—immunoglobulin G; IgM—immunoglobulin M; FCR—feed conversion ratio; *MUC2*—mucin2; *IGF-1*—insulin-like growth factor-1; AST—aspartate aminotransferase; ↑—increased; ↓—decreased.

## Data Availability

No new data were created or analyzed in this study. Data sharing is not applicable to this article.

## Abbreviations

The following abbreviations are used in this manuscript:

SBM	Soybean meal
FSBM	Fermented soybean meal
ANFs	Anti-nutritional factors
TIs	Trypsin inhibitors
NSP	Non-starchy polysaccharides
SBMP	Soybean meal protein
LPS	Lipopolysaccharide
MUN	Milk urea nitrogen
FCM	Fat-corrected milk
SCC	Somatic cell count
RUP	Rumen undegraded protein
ME	Metabolizable energy
DE	Digestible energy
CP	Crude protein
DM	Dry matter
FCR	Feed conversion rate
SBPC	Soybean protein concentrate
ADG	Average daily gain
USDA	United States Department of Agriculture
ADF	Acid detergent fiber
LAB	Lactic acid bacteria
IgA	Immunoglobulin A
IgG	Immunoglobulin G
IgM	Immunoglobulin M
ADFI	Average daily feed intake
IL-1β	Interleukin 1β
IL-6	lnterleukin 6
MUC2	Mucin2
